# A multi-scale biological network framework for discovering MiRNA-Disease associations

**DOI:** 10.3389/fbinf.2026.1873526

**Published:** 2026-07-01

**Authors:** Zhang Yu, Zuo Xuan, Tang Ying, Jiang Shanshan, Qian Kun, Li Linkai, Zhou Zihao, Hong Xiuqin, Liu Zhengyu, Pan Hongwei

**Affiliations:** 1 Cardiology Department, Hunan Provincial People’s Hospital, Changsha, China; 2 The First Affiliated Hospital of Hunan Normal University, Changsha, China; 3 School of Information Science and Engineering, Guilin University of Technology, Guilin, China; 4 Cardiovascular Epidemiology Research Laboratory, Hunan Provincial People’s Hospital, Changsha, China

**Keywords:** biological graphs, deep learning, meta-learning, miRNA-disease, multi-scale

## Abstract

**Introduction:**

Identifying potential miRNA–disease associations is essential for clarifying the molecular basis of complex diseases and accelerating the discovery of diagnostic biomarkers and therapeutic targets. However, the performance of existing computational methods is often limited by sparse biological interaction networks, highly imbalanced disease distributions, and the small number of experimentally validated associations.

**Methods:**

To address these challenges, we propose MMAG, a novel framework that formulates miRNA–disease association prediction as a meta-conditional distribution alignment problem on multi-scale biological graphs. MMAG integrates three complementary components. First, a multi-scale representation learning module captures hierarchical biological information from local topological connectivity, mesoscopic functional organization, and global spectral structure. Second, a meta-learning strategy models each disease as an individual task, enabling the model to learn disease-specific prototype representations from support samples and adapt effectively to few-shot settings. Third, a conditional adversarial alignment mechanism reduces feature distribution discrepancies across diseases with different data scales, thereby enhancing cross-task knowledge transfer and generalization.

**Results:**

Extensive experiments demonstrate that MMAG consistently outperforms several state-of-the-art methods under few-shot, long-tailed, and cross-dataset transfer scenarios.

**Discussion:**

These results indicate that MMAG provides an effective and scalable solution for miRNA–disease association prediction and offers a promising strategy for broader biological network inference tasks.

## 1 Introduction

miRNAs serve as important post-transcriptional regulators in diverse pathological processes, including tumorigenesis, neurodegenerative diseases, immune responses, and metabolic disorders (Sheng et al., 2018; Wang et al., 2021; Wei et al., 2020). Systematic identification of miRNA–disease associations is therefore of considerable significance for elucidating disease-related molecular mechanisms and for supporting the development of precision diagnosis and targeted therapy (Yu et al., 2018). However, experimental discovery of such associations is often labor-intensive, time-consuming, and costly (Piovesan et al., 2015), which has stimulated growing interest in computational methods for miRNA–disease association (MDA) prediction. Despite substantial progress in graph-based models and deep learning techniques, real-world biological networks remain highly sparse and typically exhibit pronounced long-tailed distributions as well as marked heterogeneity across diseases (Wolfers et al., 2018). In particular, rare or emerging diseases usually have only a very limited number of confirmed associations, making robust generalization under few-shot settings a major challenge for existing approaches (Negi et al., 2021). As a result, improving prediction performance for few-shot diseases within multi-scale biological network structures has become an important problem in current MDA research.

Existing computational studies for MDA prediction can generally be grouped into two major categories. The first category is based on graph representation learning, where miRNA–disease data are modeled as heterogeneous graphs or bipartite networks and topological dependencies are captured through graph propagation mechanisms (Angles and Gutierrez, 2008). These methods usually integrate similarity networks and known association networks into a unified graph for representation learning (Yan et al., 2022; Zhou et al., 2022). For example, RWRMDA employs random walk with restart to model global diffusion patterns (Chen et al., 2012); HDMP integrates miRNA and disease similarities through multi-path propagation (Heyman et al., 2022); LRLSLDA performs label propagation based on regularized least squares (Chen and Yan, 2013); MDA-CNN extracts local structural patterns using convolutional neural networks (Chen et al., 2022); GCNMDA and GAT-based MDA introduce graph convolution and graph attention to aggregate neighborhood information (Long et al., 2020); NIMCGCN adopts a multi-channel graph convolutional architecture (Li et al., 2020); and MDA-GCN together with DANE-MDA combines deep graph convolution and autoencoder-based embedding learning (Ji et al., 2021). These methods have achieved notable success in exploiting network topology and capturing local structural characteristics as well as higher-order neighborhood information. Nevertheless, they are essentially discriminative in nature and generally rely on the implicit assumption that training and test data follow similar distributions. Consequently, they lack explicit mechanisms to cope with severe data imbalance and few-shot diseases, which limits their performance in cross-disease generalization scenarios. This limitation has motivated the exploration of more expressive deep modeling and generative learning strategies.

The second category focuses on deep representation learning and multimodal feature fusion, aiming to improve predictive performance through more flexible neural architectures. Representative examples include DeepMDA and MDHGI, which combine autoencoders with graph embeddings for feature integration (Fu and Peng, 2017); MDA-Transformer, which employs self-attention to capture long-range dependencies (Zouhaier et al., 2014); and HGIMDA, which leverages heterogeneous graph information propagation (Chen et al., 2016). More recently, generative models such as variational autoencoders (VAE) (Bank et al., 2023) and generative adversarial networks (GAN) (Goodfellow et al., 2020) have also been introduced into MDA prediction, with methods such as MDA-GAN attempting to generate latent associations for data augmentation. Although these approaches improve model expressiveness and feature integration, most of them still rely on single-scale topological modeling and do not provide a systematic characterization of hierarchical biological network structures spanning local interactions, functional modules, and global topological organization. In addition, current generative methods are mainly used for sample augmentation, rather than for explicitly modeling cross-disease distribution alignment or controlling generalization error from a theoretical perspective (Ding et al., 2019). Therefore, integrating multi-scale structural modeling with meta-learning for few-shot tasks, while introducing adversarial learning to achieve explicit distribution alignment, remains a relatively unexplored but highly promising direction.

**FIGURE 1:**
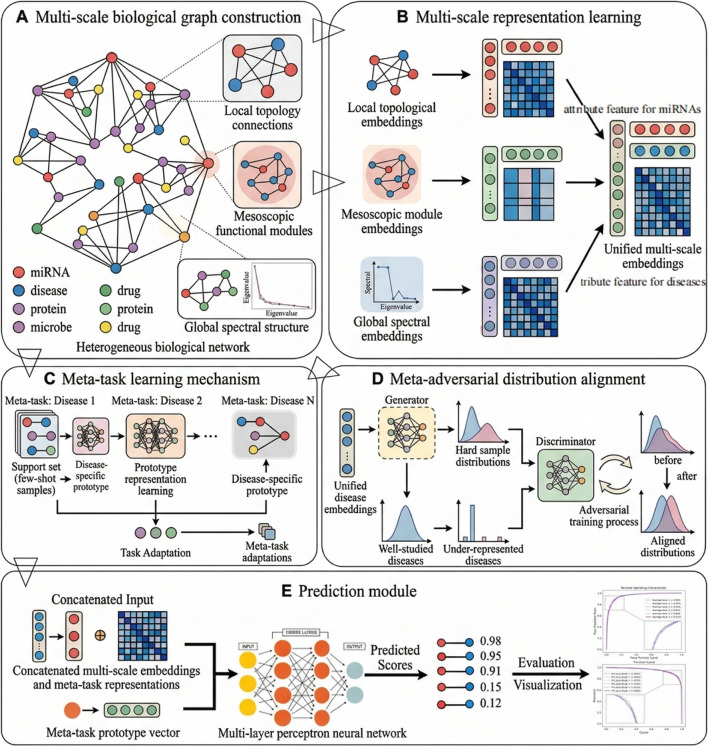
Overview of the proposed multi-scale meta-adversarial graph framework (MMAG) for miRNA–disease association prediction. **(A)** A heterogeneous biological network is constructed and characterized from three structural perspectives: local topology, mesoscopic functional modules, and global spectral structure. **(B)** Multi-scale representation learning extracts hierarchical embeddings from these levels and integrates them into unified representations for miRNAs and diseases. **(C)** A meta-task learning mechanism treats each disease as an independent task and learns disease-specific prototype representations from few-shot support sets. **(D)** A meta-adversarial strategy aligns feature distributions between well-studied and under-represented diseases through generator–discriminator training. **(E)** The fused embeddings and meta-task representations are fed into a multilayer perceptron to predict miRNA–disease associations and generate evaluation results.

To address these limitations, we reformulate miRNA–disease association prediction as a meta-conditional distribution alignment problem on multi-scale biological graphs and propose a novel multi-scale meta-adversarial graph generative framework, termed MMAG (see Figure 1). At the representation level, MMAG constructs hierarchical embeddings from three complementary perspectives, including local topological structure, mesoscopic functional modules, and global graph spectral structure. By hierarchically integrating these multi-scale features, the model is able to capture biological organization at different levels and overcome the limitations of conventional single-scale modeling. At the learning level, each disease is treated as an independent meta-task, and disease-specific prototype representations are learned from support sets to improve task adaptation and generalization in few-shot settings. Furthermore, a conditional generative adversarial mechanism is introduced, in which the generator produces hard sample distributions conditioned on disease representations, while the discriminator promotes feature distribution alignment between well-studied diseases and under-represented diseases. This adversarial design helps reduce the upper bound of cross-distribution generalization error. By jointly integrating multi-scale representation learning, meta-task optimization, and adversarial distribution alignment, MMAG establishes an end-to-end generative generalization framework for MDA prediction. Experimental results show that the proposed method consistently outperforms several state-of-the-art approaches under few-shot, long-tailed, and cross-dataset transfer settings, demonstrating its effectiveness and scalability for biological network prediction tasks.

## 2 Results

### 2.1 Performance evaluation under 5-fold cross-validation

**FIGURE 2:**
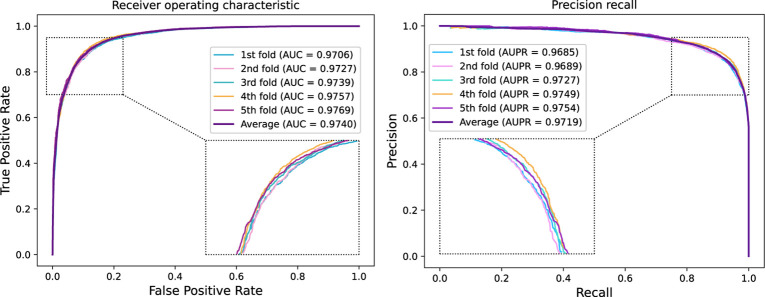
Performance evaluation of MMAG under five-fold cross-validation. Receiver Operating Characteristic (ROC) curves are shown for the five individual folds and their average curve. The Area Under the ROC Curve (AUC) values indicate the strong discriminative ability of the model across different data splits. Precision–Recall (PR) curves are shown for the corresponding five folds together with the average curve. The Area Under the Precision–Recall Curve (AUPR) reflects the balance between precision and recall and is particularly informative for imbalanced datasets. Overall, MMAG exhibits stable and consistently strong predictive performance across all folds.

To assess the generalization performance of the proposed MMAG model, we adopted five-fold cross-validation (5-fold CV) as the primary evaluation protocol. In each fold, the dataset was randomly divided into training and test subsets, allowing the model to be examined under different data partitions and thereby providing a reliable estimate of its robustness and stability. The predictive performance was further visualized using the Receiver Operating Characteristic (ROC) curves and Precision–Recall (PR) curves, as shown in Figure 2.

For quantitative evaluation, the Area Under the ROC Curve (AUC) was used as the principal metric to measure the overall discriminative capability of the model across different decision thresholds. In addition, the Area Under the Precision–Recall Curve (AUPR) was adopted to evaluate the balance between precision and recall, which is particularly informative for imbalanced data. To provide a more comprehensive assessment, the Matthews Correlation Coefficient (MCC) was also calculated. The detailed results are summarized in Table 1. Under the five-fold cross-validation setting, MMAG achieved an average AUC of 97.14%, while also maintaining consistently strong performance in terms of AUPR and MCC. These findings demonstrate that MMAG possesses reliable predictive ability and strong generalization performance for miRNA–disease association prediction.

**TABLE 1:** Performance of MMAG under five-fold cross-validation (s.t.d: standard deviation).

Fold	Accuracy	Sensitivity	Specificity	Precision	MCC	AUC
1st	0.8962	0.8895	0.9013	0.8996	0.7815	0.9692
2nd	0.8981	0.8924	0.9008	0.8978	0.7857	0.9704
3rd	0.9013	0.8947	0.9056	0.9042	0.7916	0.9715
4th	0.9072	0.9091	0.9024	0.9063	0.8042	0.9726
5th	0.9026	0.8953	0.9071	0.9021	0.7953	0.9731
Average	0.9011	0.8962	0.9034	0.9020	0.7917	0.9714
s.t.d	0.0042	0.0071	0.0035	0.0039	0.0076	0.0015

### 2.2 Parameter analysis

To further investigate the robustness of MMAG and identify suitable hyperparameter settings, we performed a parameter sensitivity analysis on two key factors: the meta-task support set size K and the multi-scale embedding dimension d . By systematically varying these parameters, we examined how task-level supervision and representation capacity affect the predictive performance of the proposed framework.

#### 2.2.1 Meta-task support set size K

To evaluate the influence of the meta-learning mechanism, we first analyzed the effect of the meta-task support set size K . In MMAG, each disease prediction task is formulated as a meta-task, and a small number of known miRNA–disease associations are used as the support set to construct disease-specific prototype representations. Therefore, the value of K directly determines how much task-specific information is available during adaptation and has a substantial impact on few-shot learning performance.

As reported in Table 2, the performance of MMAG improves steadily as K increases from 1 to 10. Even under the extremely limited setting of K=1 , the model still achieves competitive results, with an AUC of 0.9412 and an AUPR of 0.9351, indicating that MMAG can extract informative task-level representations under minimal supervision. When the support set size increases to K=3 and K=5 , all evaluation metrics show further improvement, suggesting that additional support samples provide more reliable disease-specific signals for prototype construction and representation learning. The best performance is obtained at K=10 , where MMAG achieves an AUC of 0.9623 and an AUPR of 0.9668. Overall, these results show that MMAG remains stable across different support set sizes and benefits from richer task-specific information, highlighting its effectiveness in few-shot miRNA–disease association prediction.

**TABLE 2:** Performance analysis with different meta-task support set sizes K .

Support set size K	Accuracy	Precision	Recall	F1-score	AUC	AUPR
K=1	0.8742	0.8729	0.8753	0.8741	0.9412	0.9351
K=3	0.8851	0.8836	0.8864	0.8850	0.9464	0.9423
K=5	0.8889	0.8871	0.8895	0.8883	0.9516	0.9496
K=10	**0.8927**	**0.8908**	**0.8936**	**0.8922**	**0.9623**	**0.9668**

Bold values indicate the best performance for each evaluation metric.

#### 2.2.2 Multi-scale embedding dimension d

We further evaluated the effect of the multi-scale embedding dimension d on model performance. In MMAG, node representations are learned by integrating biological information from local topology, functional modules, and global graph structure. The embedding dimension determines the representational capacity of these features and therefore directly affects the ability of the model to capture complex miRNA–disease relationships. A dimension that is too small may fail to encode sufficient structural information, whereas an excessively large dimension may introduce redundancy and increase the risk of overfitting.

The results are presented in Table 3. As d increases from 16 to 128, the predictive performance consistently improves across all metrics. When d=16 , the model already achieves an AUC of 0.9387 and an AUPR of 0.9314, indicating that low-dimensional representations can still capture essential structural patterns in the biological network. Increasing the dimension to d=32 and d=64 further improves performance, suggesting that richer representations enable the model to better characterize the interactions between miRNAs and diseases. The best results are obtained at d=128 , where MMAG achieves an AUC of 0.9723 and an AUPR of 0.9668. When the dimension is further increased to d=256 , the performance decreases slightly, which may be attributed to higher model complexity and the introduction of redundant features. These observations suggest that d=128 provides an appropriate balance between expressive power and generalization.

**TABLE 3:** Performance analysis with different multi-scale embedding dimensions d .

Multi-scale embedding dimension d	Accuracy	Precision	Recall	F1-score	AUC	AUPR
d=16	0.8642	0.8619	0.8661	0.8640	0.9387	0.9314
d=32	0.8785	0.8753	0.8801	0.8777	0.9472	0.9416
d=64	0.8859	0.8824	0.8871	0.8847	0.9536	0.9491
d=128	**0.8927**	**0.8908**	**0.8936**	**0.8922**	**0.9723**	**0.9668**
d=256	0.8874	0.8841	0.8890	0.8865	0.9587	0.9523

Bold values indicate the best performance for each evaluation metric.

### 2.3 Ablation study

To further verify the effectiveness of the major components in MMAG, we conducted ablation experiments by systematically removing key modules from the full framework. Specifically, two simplified variants were considered: (1) *MMAG_w/o_multi_scale*, in which the multi-scale representation learning module was removed, and (2) *MMAG_w/o_meta*, in which the meta-learning component was excluded while the remaining modules were retained. By comparing these variants with the complete MMAG model, we aimed to quantify the contribution of each component to the overall predictive performance.

**FIGURE 3:**
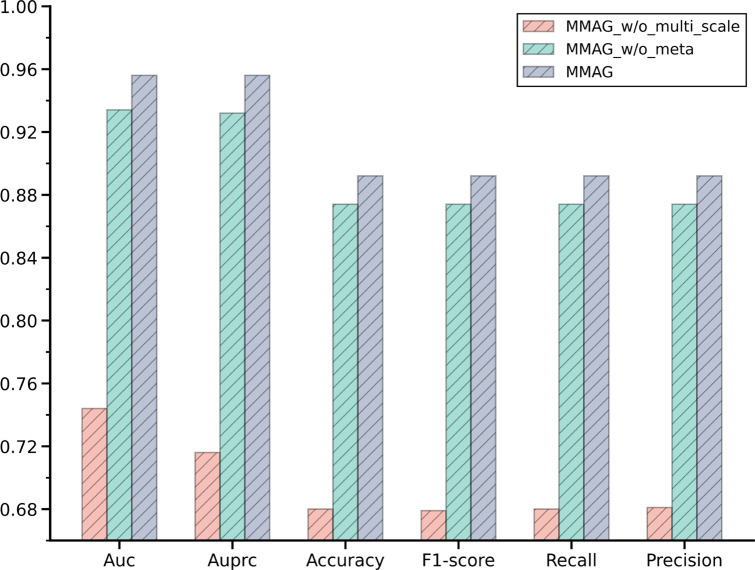
Ablation study of MMAG on the miRNA–disease association prediction task. Three model variants are compared: MMAG without the multi-scale representation module (MMAG_w/o_multi_scale), MMAG without the meta-task learning mechanism (MMAG_w/o_meta), and the full MMAG model. Performance is evaluated using AUC, AUPR, Accuracy, F1-score, Recall, and Precision. The results show that removing either the multi-scale representation module or the meta-learning component leads to a clear decline in predictive performance, confirming that both modules make substantial contributions to the full framework.

The results are shown in Figure 3. The complete MMAG model consistently achieves the best performance across all evaluation metrics, including AUC, AUPR, Accuracy, F1-score, Recall, and Precision. When the multi-scale representation module is removed, the model performance decreases markedly across all metrics, especially in terms of AUC and AUPR. This finding indicates that multi-scale structural information is essential for characterizing the complex interaction patterns embedded in the miRNA–disease biological network. Without integrating information from different structural levels, the model is unable to fully exploit the heterogeneous biological relationships.

A similar but slightly smaller performance degradation is observed when the meta-learning component is removed. This result suggests that the meta-task learning mechanism contributes substantially to cross-disease generalization by enabling the model to learn disease-specific representations from limited support samples. Taken together, the ablation results demonstrate that both the multi-scale representation module and the meta-learning mechanism are critical to the overall effectiveness of MMAG.

### 2.4 Performance comparison with state-of-the-art methods

To comprehensively evaluate the effectiveness of MMAG, we compared it with several representative state-of-the-art (SOTA) methods for miRNA–disease association prediction, including PATMDA (Xie et al., 2023), NIMCGCN (Li et al., 2020), ERMDA (Dai et al., 2022), AGAEMD (Zhang et al., 2022), and MAGCN (Lu et al., 2023). These baseline methods cover different modeling paradigms, including graph-based representation learning and deep neural network approaches, thus providing a strong basis for performance comparison. Multiple evaluation metrics, including Accuracy, Precision, Recall, F1-score, AUC, and AUPR, were adopted to ensure a comprehensive assessment.

The quantitative results are summarized in Table 4. MMAG consistently achieves the best performance across all evaluation metrics. In particular, it attains an AUC of 0.9759 and an AUPR of 0.9751, outperforming all competing methods by a clear margin. Compared with the strongest baseline, PATMDA, MMAG improves the AUC from 0.9347 to 0.9759 and the AUPR from 0.9374 to 0.9751, indicating a substantial enhancement in ranking ability for identifying potential miRNA–disease associations. Similar improvements are observed in the classification-based metrics. For example, MMAG achieves an Accuracy of 0.9324 and an F1-score of 0.9324, both of which are markedly higher than those of the compared methods.

**TABLE 4:** Performance comparison between MMAG and state-of-the-art methods.

Methods	Accuracy	Precision	Recall	F1-score	AUC	AUPR
PATMDA	0.8582	0.8599	0.8702	0.8498	0.9347	0.9374
NIMCGCN	0.8131	0.8076	0.8220	0.8148	0.8945	0.8926
ERMDA	0.8399	0.8412	0.8382	0.8396	0.9171	0.9173
AGAEMD	0.8502	0.8481	0.8544	0.8507	0.9270	0.9286
MAGCN	0.8483	0.8533	0.8425	0.8473	0.9245	0.9268
MMAG	0.9324	0.9298	0.9351	0.9324	0.9759	0.9751

The superior performance of MMAG can be attributed to its integrated framework design. The multi-scale representation learning module enables the model to capture complementary structural patterns from different levels of the biological network, while the meta-learning mechanism improves adaptability to sparse and heterogeneous disease prediction tasks. By jointly incorporating these components, MMAG learns more informative representations and exhibits stronger generalization ability than existing methods.

### 2.5 Case study

To further examine the practical predictive ability of MMAG, we performed case studies on breast cancer and colon cancer, two representative malignancies with substantial clinical relevance. For each disease, all known miRNA–disease associations were removed from the training set, and the trained model was then used to rank candidate miRNAs according to their predicted association scores. The top 50 predicted miRNAs were subsequently validated using two widely used databases, namely, dbDEMC and miRCancer.

#### 2.5.1 Breast cancer

Breast cancer is one of the most common malignancies among women worldwide and remains a major cause of cancer-related mortality. Accumulating evidence has shown that miRNAs play key regulatory roles in tumor initiation, progression, and metastasis through post-transcriptional regulation of gene expression. Therefore, identifying novel miRNA–breast cancer associations is important for elucidating disease mechanisms and for discovering potential diagnostic biomarkers and therapeutic targets.

As summarized in Table 5 47, of the top 50 miRNAs predicted by MMAG have already been confirmed in dbDEMC or miRCancer, while only three remain unverified. Several highly ranked candidates, including hsa-mir-486-5p, hsa-mir-20b-5p, and hsa-mir-125b-5p, have been reported to be closely associated with breast cancer progression and tumor-related regulatory processes. The high validation rate indicates that MMAG can effectively capture biologically meaningful associations between miRNAs and breast cancer.

**TABLE 5:** The top 50 verified associations associated with Breast Cancer.

Rank	miRNAs	Evidence	Rank	miRNAs	Evidence
1	hsa-mir-181a-2-3p	dbDEMC	26	hsa-mir-3611	dbDEMC
2	hsa-mir-486-5p	dbDEMC	27	hsa-mir-4290	dbDEMC
3	hsa-mir-20b-5p	dbDEMC	28	hsa-mir-612	dbDEMC; miRCancer
4	hsa-mir-330-5p	dbDEMC	29	hsa-mir-105-5p	dbDEMC
5	hsa-mir-452-5p	dbDEMC	30	hsa-mir-3620-5p	dbDEMC
6	hsa-mir-593-5p	dbDEMC	31	hsa-mir-340-5p	dbDEMC
7	hsa-mir-503-5p	dbDEMC	32	hsa-mir-576-5p	dbDEMC
8	hsa-mir-718	dbDEMC	33	hsa-mir-626	dbDEMC
9	hsa-mir-154-5p	dbDEMC	34	hsa-mir-519c-5p	dbDEMC
10	hsa-mir-125b-5p	dbDEMC	35	hsa-mir-136-5p	dbDEMC
11	hsa-mir-483-5p	dbDEMC	36	hsa-mir-1275	dbDEMC
12	hsa-mir-204-5p	dbDEMC	37	hsa-mir-212-5p	dbDEMC
13	hsa-mir-632	dbDEMC	38	hsa-mir-517c-3p	dbDEMC
14	hsa-mir-1246	dbDEMC	39	hsa-mir-187-5p	dbDEMC
15	hsa-mir-377-5p	dbDEMC	40	hsa-mir-202-5p	dbDEMC
16	hsa-mir-449b-5p	dbDEMC	41	hsa-mir-449a	dbDEMC
17	hsa-mir-129-2-3p	dbDEMC	42	hsa-mir-764	dbDEMC
18	hsa-mir-583	dbDEMC	43	hsa-mir-542-5p	dbDEMC; miRCancer
19	hsa-mir-1825	dbDEMC	44	hsa-mir-4487	dbDEMC
20	hsa-mir-185-5p	dbDEMC	45	hsa-mir-3151-5p	dbDEMC
21	hsa-mir-491-5p	dbDEMC	46	hsa-mir-511-5p	dbDEMC
22	hsa-mir-636	dbDEMC	47	hsa-mir-1284	dbDEMC
23	hsa-mir-885-5p	dbDEMC	48	hsa-mir-4716	unconfirmed
24	hsa-mir-3651	dbDEMC	49	hsa-mir-3665	unconfirmed
25	hsa-mir-1973	dbDEMC	50	hsa-mir-4782	unconfirmed

#### 2.5.2 Colon cancer

Colon cancer is one of the most prevalent gastrointestinal malignancies worldwide and represents a major cause of cancer-related morbidity and mortality. Increasing evidence suggests that dysregulated miRNAs are critically involved in tumorigenesis, metastasis, and therapeutic response through the modulation of oncogenic and tumor-suppressive pathways. Thus, identifying potential miRNA–colon cancer associations is essential for understanding the molecular basis of the disease and for discovering candidate biomarkers and therapeutic targets.

As shown in Table 6, 48 of the top 50 miRNAs predicted for colon cancer have already been supported by dbDEMC or miRCancer, whereas only two remain unverified. Several highly ranked candidates, such as hsa-mir-16-5p, hsa-mir-29c-5p, hsa-mir-34a-5p, and hsa-mir-200c-5p, have been widely reported to participate in colorectal tumor progression and regulatory signaling pathways. The validation ratio of 96% further demonstrates that MMAG is capable of identifying biologically relevant miRNA–disease associations from heterogeneous network data.

**TABLE 6:** The top 50 verified miRNAs associated with Colon Cancer.

Rank	miRNAs	Evidence	Rank	miRNAs	Evidence
1	hsa-mir-16-5p	dbDEMC; miRCancer	26	hsa-mir-642a-5p	dbDEMC
2	hsa-mir-29c-5p	dbDEMC	27	hsa-mir-658-5p	dbDEMC
3	hsa-mir-10a-5p	dbDEMC	28	hsa-mir-663a	dbDEMC
4	hsa-let-7g-5p	dbDEMC	29	hsa-mir-769-5p	dbDEMC
5	hsa-let-7i-5p	dbDEMC	30	hsa-mir-302c-5p	dbDEMC
6	hsa-mir-181b-5p	dbDEMC	31	hsa-mir-199a-5p	dbDEMC
7	hsa-mir-16-2-3p	dbDEMC	32	hsa-mir-378a-5p	dbDEMC
8	hsa-mir-127-5p	dbDEMC	33	hsa-mir-575	dbDEMC
9	hsa-mir-145-5p	dbDEMC	34	hsa-mir-27b-5p	dbDEMC
10	hsa-mir-150-5p	dbDEMC	35	hsa-mir-451a	dbDEMC
11	hsa-mir-30c-5p	dbDEMC	36	hsa-mir-30a-5p	dbDEMC; miRCancer
12	hsa-mir-222-5p	dbDEMC	37	hsa-mir-106b-5p	dbDEMC
13	hsa-mir-20a-5p	dbDEMC	38	hsa-mir-107	dbDEMC; miRCancer
14	hsa-mir-200c-5p	dbDEMC	39	hsa-mir-21-5p	dbDEMC
15	hsa-mir-34a-5p	dbDEMC	40	hsa-mir-214-5p	dbDEMC
16	hsa-mir-296-5p	dbDEMC	41	hsa-mir-142-5p	dbDEMC
17	hsa-mir-30d-5p	dbDEMC	42	hsa-mir-330-5p	dbDEMC
18	hsa-mir-23a-5p	dbDEMC	43	hsa-mir-452-5p	dbDEMC
19	hsa-mir-365b-5p	dbDEMC	44	hsa-mir-509-3-5p	unconfirmed
20	hsa-mir-484	dbDEMC	45	hsa-mir-4728-5p	dbDEMC
21	hsa-mir-486-5p	dbDEMC	46	hsa-mir-593-5p	dbDEMC
22	hsa-mir-511-5p	dbDEMC	47	hsa-mir-24-1-5p	dbDEMC
23	hsa-mir-520c	dbDEMC; miRCancer	48	hsa-mir-101-2-5p	unconfirmed
24	hsa-mir-615-5p	dbDEMC	49	hsa-mir-29b-1-5p	dbDEMC
25	hsa-mir-629-5p	dbDEMC	50	hsa-mir-424-5p	dbDEMC

## 3 Conclusion

In this study, we proposed a novel framework, termed MMAG, for miRNA–disease association prediction to address the challenges posed by sparse biological networks, heterogeneous disease distributions, and the limited availability of labeled associations. By formulating the prediction task as a meta-conditional distribution alignment problem on multi-scale biological graphs, MMAG unifies multi-scale representation learning, meta-learning, and adversarial distribution alignment within a single framework.

Specifically, the multi-scale representation module enables the model to characterize biological network structures from complementary perspectives, including local topological interactions, mesoscopic functional modules, and global graph organization. The meta-learning strategy further improves adaptability by treating each disease as an individual task and constructing disease-specific prototype representations, which is particularly beneficial in few-shot and long-tailed settings. In addition, the adversarial alignment mechanism reduces feature distribution discrepancies across diseases with different levels of data availability, thereby enhancing the generalization capability of the model.

Extensive experimental results demonstrate that MMAG consistently outperforms several state-of-the-art methods across multiple evaluation metrics. The parameter sensitivity analysis confirms the robustness of the proposed framework under different hyperparameter settings, while the ablation study verifies the contribution of each major component. Moreover, case studies on breast cancer and colon cancer show that most of the top-ranked predicted miRNAs are supported by existing biological databases, further highlighting the practical value of MMAG in identifying biologically meaningful miRNA–disease associations.

In future work, additional biological information, such as gene expression profiles, protein-protein interaction networks, and functional pathway data, could be incorporated to further enhance the representational capacity of the model. In addition, the integration of more advanced graph learning strategies and experimental validation pipelines may improve the biological interpretability of the predictions and provide deeper insights into the molecular mechanisms underlying complex diseases.

## 4 Methods

We formulate miRNA–disease association (MDA) prediction as a meta-conditional distribution alignment problem on a multi-scale biological graph. Given a heterogeneous graph $G=\left(V_{m},V_{d},E\right)$ , where $V_{m}$ and $V_{d}$ denote the sets of miRNA and disease nodes, respectively, and E represents the set of known miRNA–disease associations, our objective is to learn a predictive model with strong generalization capability for few-shot and under-represented diseases.

### 4.1 Multi-scale representation learning

Biological networks are inherently organized in a hierarchical manner, involving local interaction patterns, mesoscopic functional modules, and global structural distributions. Relying solely on single-scale graph modeling is often insufficient to capture these complementary biological characteristics. To address this issue, we construct multi-scale node representations to encode structural information from different organizational levels.

#### 4.1.1 Local-scale encoding

To characterize immediate interaction patterns between miRNAs and diseases, we employ a graph attention encoder on the bipartite association graph, as defined in Equation 1:

$$h_{i}^{\left(l+1\right)}=\sigma \left(\underset{j\in N\left(i\right)}{\sum }\alpha_{ij}W_{l}h_{j}^{\left(l\right)}\right),$$ (1)

where N(i) denotes the neighbors of node i , $\alpha_{ij}$ is the attention coefficient, and $W_{l}$ is the trainable weight matrix. The resulting local embedding is denoted as $z_{i}^{\mathrm{local}}$ .

#### 4.1.2 Mesoscopic module encoding

To capture functional similarity among miRNAs and diseases, we further construct group-level prototype representations based on similarity graphs. Specifically, the mesoscopic representation of each functional module is defined as the average of local-scale embeddings of all nodes within that module, as shown in Equation 2:

$$z_{g}^{\mathrm{meso}}=\frac{1}{\left|V_{g}\right|}\underset{i\in V_{g}}{\sum }z_{i}^{\mathrm{local}},$$ (2)

where $V_{g}$ denotes the set of nodes belonging to the same functional module. Each node is then assigned a mesoscopic embedding according to its module membership.

#### 4.1.3 Global spectral encoding

To incorporate global topological information, we introduce a spectral graph encoding scheme based on the normalized Laplacian matrix in Equation 3:

$$L=I-D^{-\frac{1}{2}}AD^{-\frac{1}{2}},$$ (3)

where A is the adjacency matrix and D denotes the corresponding degree matrix. Global structural features are then extracted through eigen-decomposition of L , as shown in Equation 4:

$$z_{i}^{\mathrm{global}}=U_{k}\left(i\right),$$ (4)

where $U_{k}$ contains the top- k eigenvectors, and $U_{k}\left(i\right)$ denotes the spectral representation of node i .

#### 4.1.4 Multi-scale fusion

The final node representation is obtained by adaptively integrating the embeddings from different structural scales, as formulated in Equation 5:

$$z_{i}=\alpha z_{i}^{\mathrm{local}}+\beta z_{i}^{\mathrm{meso}}+\gamma z_{i}^{\mathrm{global}},$$ (5)

where α , β , and γ are learnable fusion coefficients.

### 4.2 Meta-prototype learning

Rare and emerging diseases usually have only a small number of confirmed associations. To improve model adaptation under few-shot settings, we regard each disease as an individual meta-task and learn disease-specific prototype representations.

For each disease d , we define a task $T_{d}=\left(S_{d},Q_{d}\right)$ , where $S_{d}$ and $Q_{d}$ denote the support set and query set, respectively.

#### 4.2.1 Disease prototype construction

For a given disease d , its prototype representation is computed by averaging the embeddings of miRNAs in the corresponding support set, as shown in Equation 6:

$$p_{d}=\frac{1}{\left|S_{d}\right|}\underset{m\in S_{d}}{\sum }z_{m}.$$ (6)

#### 4.2.2 Association prediction

Given a miRNA m and a disease d , the association score is estimated using Equation 7 as

$$s\left(m,d\right)=\sigma \left(z_{m}^{⊤}W_{p}p_{d}\right),$$ (7)

where $W_{p}$ is a learnable parameter matrix and σ(⋅) denotes the sigmoid activation function.

The meta-task optimization objective is defined in Equation 8 as

$$L_{\mathrm{meta}}=-\underset{\left(m,d\right)\in Q_{d}}{\sum }\left[y_{md}\log s\left(m,d\right)+\left(1-y_{md}\right)\log\left(1-s\left(m,d\right)\right)\right].$$ (8)

### 4.3 Adversarial distribution alignment

The distribution of disease-associated samples is typically highly imbalanced. As a consequence, models trained predominantly on well-studied diseases often show limited generalization ability when applied to under-represented diseases. To mitigate this cross-disease distribution discrepancy, we introduce a conditional adversarial alignment mechanism.

#### 4.3.1 Conditional generator

The generator produces synthetic embeddings conditioned on the disease prototype, as described in Equation 9:

$$\tilde{z}=G\left(p_{d},\epsilon \right),$$ (9)

where ϵ∼N(0,I) denotes Gaussian noise.

#### 4.3.2 Discriminator

The discriminator aims to distinguish real embeddings from generated ones using Equation 10:

$$D\left(z\right)=\sigma \left(w_{d}^{⊤}z\right).$$ (10)

Accordingly, the adversarial learning objective is formulated in Equation 11 as

$$L_{\mathrm{adv}}=E_{z∼P_{\mathrm{real}}}\left[\log D\left(z\right)\right]+E_{\tilde{z}∼P_{G}}\left[\log\left(1-D\left(\tilde{z}\right)\right)\right].$$ (11)

### 4.4 Unified objective function

The overall training objective jointly incorporates the meta-learning loss, adversarial distribution alignment, and cross-scale consistency regularization. To encourage consistency between local and global structural representations, we define the regularization term in Equation 12

$$L_{\mathrm{cons}}=\underset{i}{\sum }{\left\|z_{i}^{\mathrm{local}}-z_{i}^{\mathrm{global}}\right\|}_{2}^{2}.$$ (12)

The final objective function is given by Equation 13:

$$L=L_{\mathrm{meta}}+\lambda_{1}L_{\mathrm{adv}}+\lambda_{2}L_{\mathrm{cons}},$$ (13)

where $\lambda_{1}$ and $\lambda_{2}$ are hyperparameters that control the contributions of adversarial alignment and consistency regularization, respectively.

The overall optimization process can be expressed as the minimax problem in Equation 14:

$$\underset{\Theta_{G},\Theta_{E}}{\min}\underset{\Theta_{D}}{\max}L.$$ (14)

## Data Availability

The original contributions presented in the study are included in the article/supplementary material, further inquiries can be directed to the corresponding authors.
